# Glucagonoma Associated with Calculous Pancreatitis

**DOI:** 10.1155/1996/79601

**Published:** 1996

**Authors:** Chuanyan Mao, Parviz M. Pour, John M. Howard

**Affiliations:** 1 The Departments of Surgery Toledo Hospital and Medical College of Ohio Toledo Ohio USA; 2 The Eppley Institute for Research in Cancer University of Nebraska Omaha Nebraska USA


*HPB Surgery*, 1996, Vol. 9, pp. 77–81
Reprints available directly from the publisher
Photocopying permitted by license only

© 1996 OPA (Overseas Publishers Association)
Amsterdam B.V. Published in The Netherlands
by Harwood Academic Publishers GmbH
Printed in Malaysia

### Glucagonoma Associated with Calculous Pancreatitis

CHUANYAN MAO,* PARVIZ M. POUR,** and JOHN M. HOWARD*

*The Departments of Surgery, Toledo Hospital and Medical College of Ohio, Toledo, Ohio, USA
**The Eppley Institute for Research in Cancer University of Nebraska, Omaha, Nebraska, USA

### INTRODUCTION

Glucagonomas are rare, constituting only about 6% of all islet cell tumors[1, 2]. The incidence of islet cell tumors associated with pancreatitis is apparently much lower than that of pancreatic exocrine tumors[3–5], only 13 patients currently being identified in the literature[6–16]. Although aware of another patient with a glucagonoma associated with chronic pancre atitis[17], we currently find no report of such an association in the literature. Because of its rarity and its possbile implications, the observations of a pancreatic glucagonoma association with acute and chronic pancreatitis with pancreatic lithiasis seem worthy of presentation.

### SUMMARY

A Patient with a glucagonoma associated with calculous pancreatitis is reported. The authors know of only one other such patient. Because the calculous pancreatitis developed both upstream and downstream from the tumor, it cannot be attributed to obstruction of the pancreatic duct by the tumor. Continued critical appraisal is needed to determine whether the association of the glucagonoma and pancreatitis is etiologically related or merely coincidental.

*Address for correspondence:* John M. Howard, M.D., Medical College of Ohio P.O. Box 10008 Toledo. Ohio, 43 699 USA. Phone: (419) 381-4921, Fax (419)381-3057

### CASE REPORT

A 49 year-old white female business executive was rehospitalized with a 20 month history of recurrent attacks of acute pancreatitis. During the last eight months her abdominal pain had been severe, refractory and typical of chronic pancreatitis. Detailed exploration of her personal and familial history, as well as multiple biochemical and hematological profiles, including normal serum total protein, albumin, calcium, lipids, and glucose, revealed no suggestion of any etiological factor. She had no gallstones and had neither diabetes nor steatorrhea. There was no history of renal calculi nor hyperpara-thyroidism. Physical examination revealed a slender, energetic, intelligent woman with tenderness across the upper abdomen. Her skin and tongue were normal.

CT scan and plain roentgenograms, showed pancreatic calcification, but no tumor in the head of the pancreas (Fig. 1). ERCP revealed a pancreatic duct which was dilated throughout its entire course (Fig. 2), no obstruction of the duct being detected. Longitudinal pancreaticojejunostomy was elected for interruption of her pancreatic pain.

At laparotomy, February 4, 1992, the pancreatic duct (1.0–1.3 cm diameter) was incised longitudinally in the body and head of the gland. The gland was atrophic, firm, and typical of advanced chronic pancreatitis. Numerous intraductal calculi were present in the head and body. In the uncinate process, an irregularly surfaced, white stone branched into the small ductal tributaries. All recognizable stones were

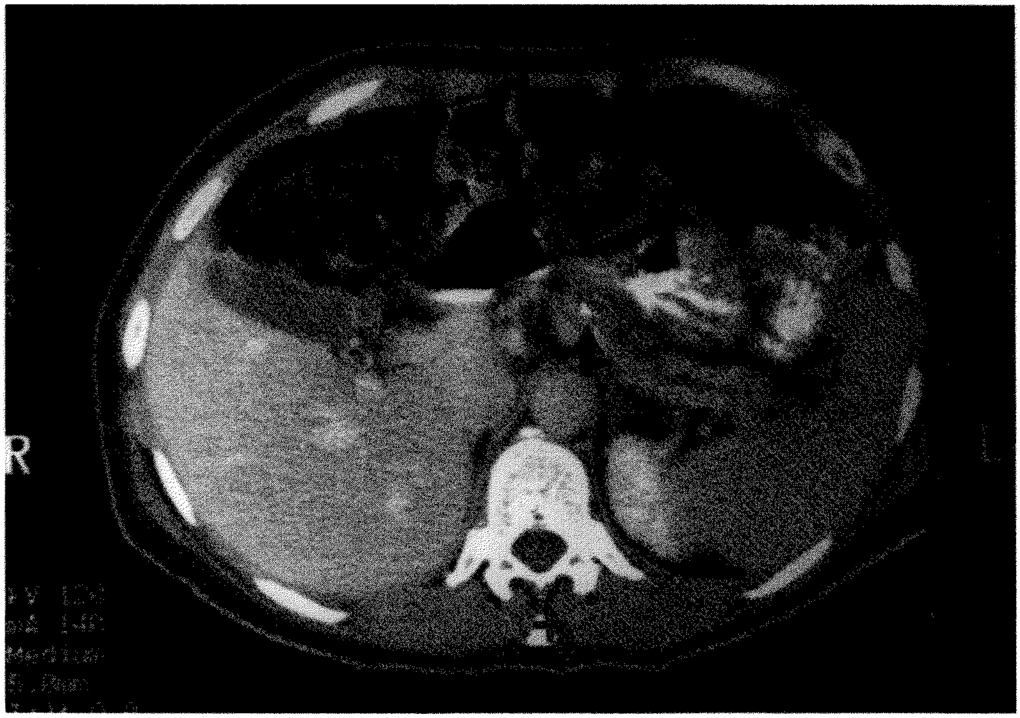

**Figure 1** CT scan–stones (solid arrow) and the dilated pancreatic duct are observed.

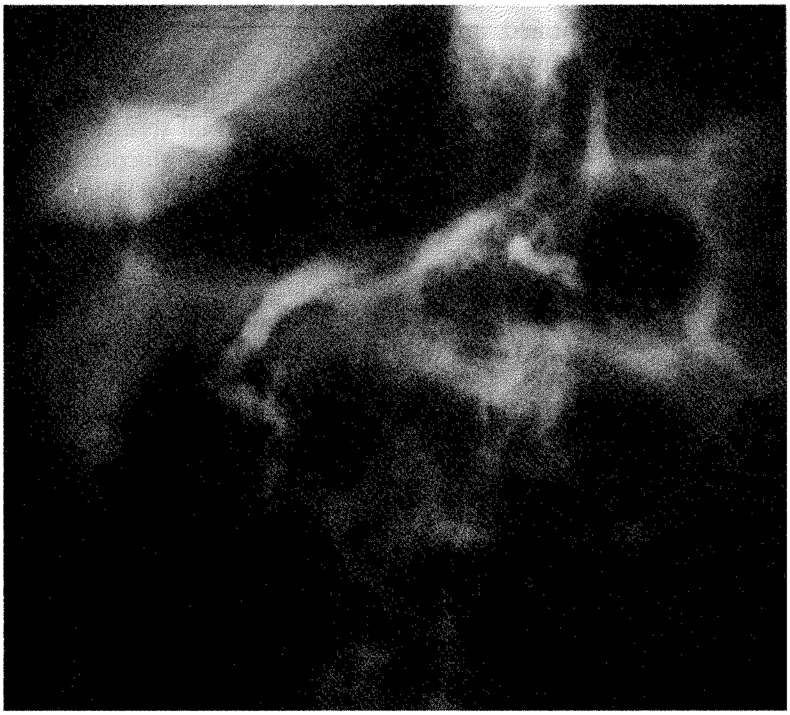

**Figure 2** ERCP shows the dilation of the pancreatic duct through the pancreas (arrow) and the stones in the small ducts of the uncinate process (solid arrow).

removed. Meanwhile, it had become apparent that the incision had transected a 1.0–1.5 cm pancreatic tumor overlying, but neither invading nor obstructing, the duct in the neck of the pancreas. Frozen section was reported as demonstrating a malignant tumor, "probably acinar cell" in type. Second and third opinions by pathologists concurred. A radical resection of the head and body of the pancreas, including skeletonization of the adjacent vessels, was performed. Histologically, the pancreas revealed atrophy of the acini with extensive fibrosis. The tumor consisted of small cell clusters scattered within a dense connective tissue. Most tumor cells were reactive with anti-glucagon antibody (Fig. 3) and only a few with anti-insulin, or anti-somatostatin. No reactivity was seen with anti-pancreatic polypeptide. In some areas, tumor cells, unreactive with any of the above antibodies, formed tubular or glandular structures (Fig. 4). Despite their apparent diffuse growth, no vascular nor neural invasion could be identified.

The patient's convalescence was uneventful. Over the ensuing three and a half years, she has remained entirely well. Her blood glucose levels remain normal, as do her biochemical and hematological profiles. Her plasma glucagon level is normal and repeated CT scans of the abdomen and chest x-rays reveal no evidence of metastasis.

### DISCUSSION

In 1942, Becker *et al.*[18] reported an association between a pancreatic tumor and a peculiar dermatitis in a patient with diabetes, weight loss and anemia, but the glucagonoma was not well recognized until 1966 when McGravan *et al.*[19] documented the presence of elevated glucagon levels in the blood and tumor tissue of a patient with an alpha-cell pancreatic neoplasm. To date, approximately 200 glucagonomas have been reported[20, 21]. Most patients have presented, in varying degrees, with the "glucagonoma syndrome" (i.e. hypoaminoacidemia, anemia, glucose intolerance, migratory necrolytic dermatitis, weight loss, stomatitis,

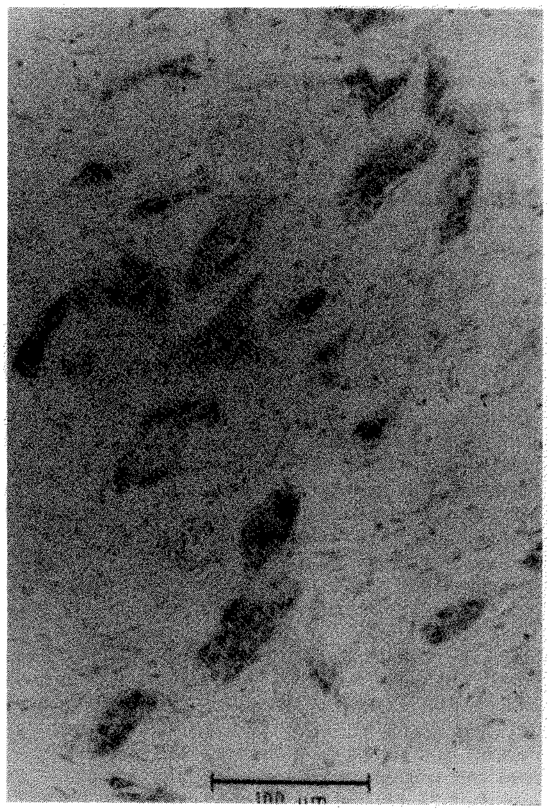

**Figure 3** Cords of tumor cells in a dense connective tissue. Most tumor cells have reacted with anti-glucagon and a few with anti-insulin or anti-somatostatin but none with anti-pancreatic polypeptide. ABC method. Bar = 100 μm.

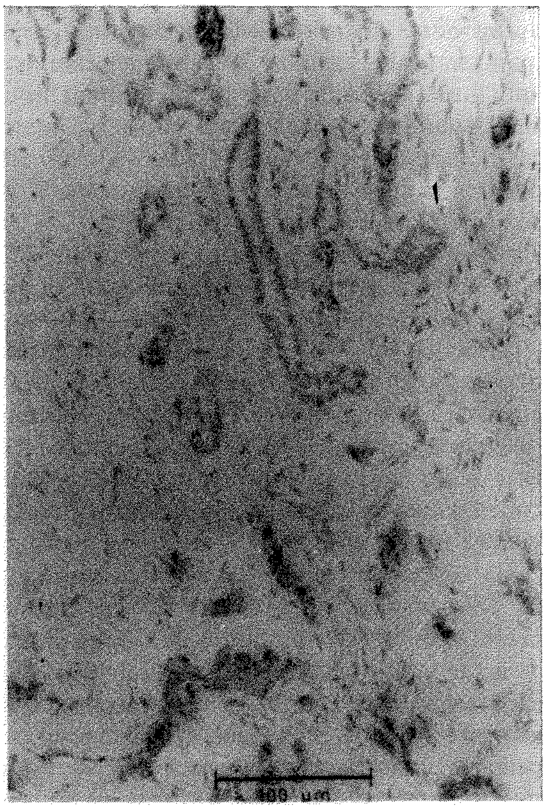

**Figure 4** Another area of the same tumor in Fig. 3. Tumor cells forming small cords have reacted with anti-glucagon, but the cells constituting the glandular structures within the tumor were unreactive with antibodies against insulin, glucagon, somatostatin or PP. Note the dense connective tissue. ABC method. Bar = 100 μm.

thromboembolism). Since the development of the radioimmunoassay and immunohistochemical staining, an increasing number of glucagonomas have been diagnosed earlier, apparently preceding the development of a "glucagonoma syndrome"[22]. Such a syndrome was not recognized, even retrospectively, in our patient. There was no glossitis, skin rash, elevated blood glucose level nor other sign of endocrine abnormality so that serum hormone levels had not been determined preoperatively. Immunohistochemical stains of the tumor demonstrate glucagon containing granules, indicative of their alpha-cell origin. Glucagonomas, as other islet cell neoplasms, may secrete multiple hormones, either entopic or ectopic, e.g. insulin, pancreatic polypeptide, somatostatin, ACTH[8], or calcitonin[23]. In the current tumor, insulin and somatostatin were detected as minor hormone products on immunohistochemical staining. It has been estimated[24] that 70% of glucagonomas are malignant and over 50% have metastasized at the time of diagnosis. So far, we recognize no stigmata of malignancy in our patient.

Glucagonoma associated with pancreatitis, especially pancreatolithiasis, must be extremely rare as we have not found such a report in the literature. Through personal communication, we are aware of another patient in whom a glucagonoma invaded and obstructed the pancreatic duct, apparently causing chronic pancreatitis upstream from the obstruction[17].

Glucagon suppresses secretion of pancreatic juice, but any mechanism of its possible induction of pancreatitis or pancreatic lithiasis is not evident. Mechanical obstruction of the pancreatic duct is usually considered the etiology of tumor-related pancreatitis, although parathyroid tumors are well recognized causes of pancreatitis and pancreatic lithiasis. Our clinical experience and a review of the literature of pancreatitis associated with islet cell tumors partially supports the obstructive mechanism[9,10,13]. On the other hand, the duct is not always obstructed. For example, Mitchenere *et al.*[6] reported a patient with an islet cell adenoma associated with acute pancreatitis, no ductal obstruction being found by pancreatography. Kaufman et al.[11] reported a tumor in the tail of the pancreas, but a pseudocyst developed in the head. In the current case, the pancreatic duct was not obstructed by the tumor; the duct was dilated and filled with calculi both upstream and downstream from the tumor.

Allison and associates[8] described the finding of calcific pancreatitis in an alcoholic patient with a malignant islet cell tumor which secreted gastrin, ACTH and apparently human chorionic gonadotrophin. Perhaps our patient had chronic idiopathic pancreatitis, by chance associated with a glucagonoma, but a cause and effect relationship remains an intriguing possibility.

The rarity of acinar cell carcinomas and their cellular appearance have previously caused them to be erroneously diagnosed as islet cell tumors–the reverse of the currrent events.

### REFERENCES

1. Klöppel, G. and Heitz, P. U. (1988) Pancreatic endocrine tumors. *Pathology, Research, and Practice*, **183**, 155–168.
2. Prinz, R.A., Sugimoto, J., Lorincz, A. L., and Kaplan E. L. (1987) Glucagonoma. In: Surgical Discases of the Pancreas. Edited by Howard, J. M., Jordan, G. L. Jr., Reber, H. A. pp. 848–859. Philadelphia: Lea & Febiger.
3. Lin, A. and Feller, E. R. (1990) Pancreatic carcinoma as a cause of unexplained pancreatitis: Report of ten cases. *Annals of Internal Medicine*, **113**, 166–167.
4. Greenberg, R. E., Bank, S., and Stark, B. (1990) Adenocarcinoma of the pancreas producing pancreatitis and pancreatic abscess. *Pancreas*, **5(1)**, 108–113.
5. Mao, C. Y., Domenico, D. R., Kim, K., Hanson, D. J., and Howard, J. M. (1995) Observations on the developmental patterns and the consequences of pancreatic exocrine adenocarcinoma based on the findings of 154 autopsies. *Archives of Surgery*, **130**, 125–134.
6. Mitchenere, P., Scott, J., and George, P. (1981) Islet cell adenoma and retention cyst: A rare cause of acute pancreatitis. *British Journal of Surgery*, **68**, 443–444.
7. Danne, P. D., Buls, J. G., Connell, J., and Bennett R. C. (1985) A case of pancreatitis associated with gastrinoma. *Australian and New Zealand Journal of Surgery*, **55**, 213–215.
8. Allison, M. C., Renfrew, C. C., Webb, W. J., Chappell, M. E., and Pounder, R. E. (1985) Case report-Neuroendocrine islet cell tumour producing gastrin and ACTH in a patient with calcifying chronic pancreatitis. *Gut*, **26**, 426–428.
9. Odaira, C., Choux, R., Payan, M. J., Bockman, D. E., and Sarles H. (1987) Chronic obstructive pancreatitis, nesidioblastosis, and small endocrine pancreatic tumor. *Digestive Disease & Sciences*, **32**, 770–774.
10. Gettenberg, G., Zimbalist, E., Marini, C. (1988) Chronic pancreatitis and pseudocyst formation secondary to carcinoid tumor of the pancreas. *Gastroenterology*, **34**, 1222–1224.
11. Kaufman, A. R., Sivak, M. C., Ferguson, D. R. (1988) Endoscopic retrograde cholangiopancreatography in pancreatic islet cell tumors. *Gastrointestinal Endoscopy*, **34**, 47–52.
12. Hanson, J. S., Cohen, A. R., Van Moore, A., Cloud, W. G., and Bianchi, R. F. (1990) Gastrinoma presenting as chronic pancreatitis. *American Journal of Gastroenterology*, **85**, 178–180.
13. Nagai, E., Yamaguchi, K., Hashimoto, H., and Sakurai T. (1992) Carcinoid tumor of the pancreas with obstructive pancreatitis. *American Journal of Gastroenterology*, **87**, 361–364.
14. Prescott, R. J., Mauson, J., and Hadondi, N. Y. (1993) Malignant islet cell tumor arising in chronic pancreatitis. *Histopathology*, **22**, 499–501.
15. Cheslyn-Curtis, S., Sitaram, V., and Williamson, R. C. N. (1993) Management of non-functioning neuroendocrine tumors of the pancreas. *British Journal of Surgery*, **30**, 625–627.
16. Broughan, T. A., Leslie, J. D., Soto, J. M., and Hermann, R. E. (1986) Pancreatic islet cell tumors. *Surgery*, **99**, 671–678.

17. Buttenschön, K., Büchler, M., Mattfeldt, T., and Beger, H. G. (1994) Glucagonoma Presenting as chronic pancreatitis. Case report and review of the literature. (Personal communication).
18. Becker, S. W. Kahn, P., and Rothman, S. (1945) Cutaneous manifestations of internal malignant tumors. *Archives of Dermatology and Syphilology*, **45**, 1068–80.
19. McGraven, M. H., Unger, R. H., Recant, L., Polk, H. C., Kilo, C., and Levin, M.E. (1966) A glucagon secreting alpha cell carcinoma of the pancreas. *New England Journal of Medicine*, **274**, 1408–1413.
20. Boden G. (1989) Glucagonoma and insulinomas. *Gastroenterology Clinics of North America*, **18**, 831–845.
21. Edney, J. A., Hofmann, S., Thompson, J. S., and Kessinger A. (1990) Glucagonoma syndrome is an underdiagnosed clinical entity. *American Journal of Surgery*, **160**, 625–629.
22. Thompson, G. B., van Heerden, J. A., Grant, C. S., Carney J. A., and Ilstrup D. M. (1988) Islet cell carcinomas of the pancreas: A twenty-year experience. *Surgery*, **104**, 1011–1017.
23. Howard, J. M., Gohara, A. F., and Cardwell, R J. (1989) Malignant islet cell tumor of the pancreas associated with high plasma calcitonin and somatostatin levels. *Surgery*, **105**, 227–229.
24. Modlin, I. M., Lewis, J. J., Ahlman, H., Bilchik A. J., and Kumar R. R. (1993) Management of unresectable malignant endocrine tumors of the pancreas. *Surgery, Gynecology & Obstetrics*, **176**, 507–518.

